# Case Department

**Published:** 1889-04

**Authors:** 


### CASE DEPATMENT.

Extraction of a Cataract Luxated into the Anterior Chamber of a Cat's Eye.—The following case may be of interest as a contribution to comparative pathology.

The patient was a fine male cat aged fourteen years. The first signs of opacity were noticed in the crystalline lens of his left eye during the fall of 1887, and soon the failure of sight in that eye was plainly noticeable. I entertained some thoughts at this time of attempting to induce absorption of the lens while it was yet soft, by means of keratonyxis, but finally abanboned them. Meanwhile the opacity grew more and more dense until after the lapse of a few months the entire lens had become a mature senile cataract. In September of the following year, 1888, the upper part of the cataract appeared to project a little way into the anterior chamber. It seemed loosened but not freely movable. But in December it fully assumed various positions, which were permitted by the great depth of the anterior chamber, corresponding to the varied positions of the head. When the cat's face looked downward the lens lay directly upon the cornea, when upward, it lay back against the iris, and could be made to assume very nearly its normal position by manipulation of the head. When the face looked forward the lens assumed a horizontal plane in the lower portion of the chamber allowing light to enter the upper half of the pupil. Finally a slight amount of reddish brown discoloration appeared near the pupillary margin of the iris, appearing like very small ciliary hemorrhages. This seemed to be due to the amount of traumatism caused by the almost continual movement of the lens, which was now acting as a foreign body in the anterior chamber, and I considered it to be the precursor of serious inflammation of the eye-ball, hence I determined to remove the lens. Some difficulty in performing the operation was anticipated on account of the great proportional size of a cat's lens as well as the still greater proportional depth of the anterior chamber and the deep set position of the eye-ball, which at the same time rendered the position of the lens after section of the cornea very uncertain and rendered the use of the bident difficult if not impossible. In view of these considerations I decided to make as large a conical flap as possible, and to extract the lens with a sharp hook. The lens presented at the opening in the best possible position and was readily extracted. A bandage was applied, and light was excluded from both eyes for four days. Healing progressed very satisfactorily. Upon removal, the lens was found to be enveloped in its capsule and seemed to have been completely detached from all of its supports. Before the operation I examined the pupils in search of a membrane and could find none, but a fortnight afterward a small amount of membrane could be seen in the form of a slight band in the outer and lower portion of the pupil near the edge of the iris.

Cataract is not infrequently met with in the eyes of all mammals, and there is no reason to believe that Nature will not here as elsewhere in the body, endeavor to so dispose of a useless or offending portion as to conserve as far as possible the functions of the organ. With a lens opaque and occupying its normal position an eye is rendered sightless, and the lens must be removed in order to obtain a certain degree of sight. Nature succeeded in doing this, in this case, to as large an extent as was possible, by gradually releasing the lens from its attachments and allowing it to fall forward into the anterior chamber where it was as much out of the way as it could be. That the integrity of the eye itself was threatened by this condition is only another evidence of the imperfect means at Nature's command, for extending portions which have come to act as foreign bodies from the tissues.

Another point worthy of notice is the fact that very little membrane has formed since the removal of the cataract. That the lens was enveloped in the capsule when removed does not, to my mind, furnish a sufficient explanation, because usually when a cataract is removed in its capsule, a membrane is found, perhaps from material exuded in consequence of the traumatism inflicted by the removal. But in this case the detachment was accomplished so slowly as not to cause any appreciable exudation, and so no visible membrane. I am inclined to ascribe the membrane now present not to the separation of the capsule from its surroundings, but to some other traumatism, possibly the same which caused the hemorrhages spoken of above, previous to the operation.

The appearance of the eye has been materially improved. It is full, round and bright. It is difficult to test the vision, but I

think it is sufficient to enable the cat to find its way about in case any misfortune should happen to his other eye.

MATTHIAS L. FOSTER, M. D.
*Clinical Assistant, Manhattan Eye and Ear Hospital, N. Y.*

CONTRIBUTION TO THE HISTORY OF CONGENITAL INTESTINAL MALFORMATION.—May 15th, 1888, I was called to see a foal twenty-four hours old, that was supposed to be suffering from constipation. The owner told me it had been foaled the morning before, had sucked and seemed as bright as any colt until about 2 P. M., when he noticed that it had colicky pains, and thinking it might be constipated he gave it a dose of Linseed Oil, repeating it in the evening. The next morning I was called and found the little animal suffering great pain. I gave oil and chloral hydrate, and applied hot rugs to the abdomen. I attempted to give an injection but could get only a small quantity of water into the rectum. I then introduced my finger but could not find anything abnormal. That evening I gave the case up as hopeless. I returned the next morning and made an autopsy. I found the rectum empty for about eight inches, and ending in a *cul. de sac.*; it had no connection with the intestines. The end of the large intestine was folded forward against the diaphragm, and was filled with fæcis.

*Chicago Veterinary College.* HALLECK S. CAMPBELL.

REPORT OF A CASE OF TUBERCULOSIS IN A DOG.*—The following case of tuberculosis in a dog illustrates the transmissibility of this disease from mankind to the lower animals.

The animal in question was a young white poodle-dog. I was first called to see him on December 29th, last, and was told that he had been sick several days, and the owner's family thought he had taken cold. When I first saw him his nose was cool and moist, the respiration rapid and jerky, somewhat abdominal. The history then was that at times his nose was hot and dry, and that his appetite was capricious, sometimes good, and at other times poor.

I thought it was a bad case of bronchitis, and prescribed a simple cough syrup, telling the people of the house to let me know if he was not better in a few days. I saw him again in much the same condition December 31st, and again January 2d.

* Read before the Massachusetts Veterinary Association.

January 2d, I learned more of the history of the case, which aided me in reaching a hasty, but, as was afterward proved, a correct diagnosis.

Mrs. H., the wife of the owner of the house where the dog was kept, died of consumption, November 8th, last, after a long and lingering illness of over a year. The dog, then a puppy, had been given to the children of the family the previous July, and I was told that he had acquired the disgusting habit of eating the sputa from the spittoon coughed up by Mrs. H., whenever he had an opportunity, although he was continually driven away when he was noticed to be indulging his abnormal appetite.

"Is it not possible," I was asked, "that he may have taken consumption from Mrs. H ?" I thought it not only possible, but probable, and as he was the pet of young children advised his speedy destruction. He was therefore shot that evening, January 2d, and I held an autopsy upon him the following morning.

The post-mortem examination revealed phthisis of both lungs, with a large abcess in the posterior lobe of the left. There was also a little puro-serous fluid in the thoracic cavity, and a somewhat fatty liver, which I thought might contain miliary tubercles.

Scrapings were made from the wall of the abcess, and smeared on cover glasses, and then stained for the bacilli of tuberculosis, which I found in large numbers under the microscope. In places there were clumps of bacilli, ten or twelve in number.

Cover glass preparations was also made from the liver, but revealed no bacilli or signs of tubercle ; they contained, however, a large number of fat cells.

Owing to lack of time, no sections have been cut for examination from either lungs or liver.

Excepting the liver, the abdominal viscera appeared healthy. I regret to say that I did not open the stomach or intestines, but they showed no outward signs of disease. This appears to be a case of inhalation tuberculosis, rather than one of ingestion, but the bacilli no doubt gained access to the respiratory apparatus as a result of the dog's filthy habits.

We often hear stories of dogs and cats kept as pets by consumptives, pining away and dying, but we do not often have a case recorded backed up by absolute proofs, where a post-mortem is held on the animal, and the bacilli found as final evidence of the disease.

*Boston, Mass.* AUSTIN PETERS, M.R.C.V.S.

Report of a Case of Molluscoid Fibromata in a Heifer.—January 31st, I went to the farm of Mr. H., at Concord, Mass., the owner having requested me to come out there, a few days previous to the above date, to see a two-year old Jersey heifer, which had a lot of "little tumors on her belly, which look more like a lot of mushrooms growing upon her" than anything he could think of.

The heifer was indeed a sight to behold. The under surface was a mass of warty-looking tumors, hanging down several inches, extending forward to within three or four inches of the fore-legs, and backward, covering the udder, and extending some distance up the escutcheon. They were pedunculated, massed closely together, and varied in size from a pea to a hen's egg. Their surface was smooth and devoid of hair, and they looked very much like a cluster of small potatoes, or, as the owner suggested, "mushrooms," depending from the ventral surface of the body. I started to count them, but as I had to take a train back to Boston, found I had not time, but estimated their number at from six to eight hundred. I removed two or three of the largest by twisting them from their pedicles. When fresh cut in two with a knife, the cut surface presented a pearly glistening appearance and a quantity of serous fluid dripped from them; at the base blood vessels entering them could be clearly seen, which in dividing and spreading through the substance of the tumor gave a very pretty injection appearance. In some of them one or two spots of extravasated blood were to be seen, due to rupture of small vessels.

Specimens of the tumors sent to the Pathological Department of the Harvard Medical School were pronounced by Dr. Sears to be molluscoid fibromata.

The growth has been very rapid, as it was not noticed until the animal was brought up from pasture last fall, and then looked like only a few small warts. A number of small warty growths had begun to appear on the under surface of the tail, on the near shoulder, and in places on both sides of the neck.

As I did not deem it worth while to try treatment, the animal was destroyed.

Austin Peters, M.R.C.V.S.

